# Expression Quantitative Trait Loci (eQTL) Mapping in Korean Patients With Crohn’s Disease and Identification of Potential Causal Genes Through Integration With Disease Associations

**DOI:** 10.3389/fgene.2020.00486

**Published:** 2020-05-14

**Authors:** Seulgi Jung, Wenting Liu, Jiwon Baek, Jung Won Moon, Byong Duk Ye, Ho-Su Lee, Sang Hyoung Park, Suk-Kyun Yang, Buhm Han, Jianjun Liu, Kyuyoung Song

**Affiliations:** ^1^Department of Biochemistry and Molecular Biology, University of Ulsan College of Medicine, Seoul, South Korea; ^2^Human Genetics, Genome Institute of Singapore, Singapore, Singapore; ^3^Department of Gastroenterology, Asan Medical Center, University of Ulsan College of Medicine, Seoul, South Korea; ^4^Department of Medicine, Seoul National University College of Medicine, Seoul, South Korea

**Keywords:** Crohn’s disease, gene expression, eQTL, Asian, blood

## Abstract

**Background:**

Expression quantitative trait loci (eQTL) datasets have extensively been used to help interpret genome-wide association study signals. Most eQTL analyses have been conducted with populations of European ancestry.

**Objective:**

To determine the most functionally relevant genes at the Crohn’s disease (CD) loci identified in genome-wide association studies (GWAS) involving Asian populations and to find novel disease-associated genes, we conducted an eQTL analysis.

**Methods:**

eQTL analysis was performed using whole-blood RNA-sequencing of 101 Korean patients with CD. FastQTL was used for a pair-wise genome analysis of ∼ 6.5 M SNPs and ∼ 22 K transcripts.

**Results:**

We identified 135,164 *cis-*eQTL and 3,816 eGenes with a false discovery rate less than 0.05. A significant proportion of the genes identified in our study overlapped with those identified in previous studies. The significantly enriched pathways of these 3,816 eGenes included neutrophil degranulation and small molecule biosynthetic process. Integrated analysis of CD GWAS with Korean eQTL revealed two putative target genes, *TNFSF15* and *GPR35*, at two previously reported loci, whereas *TNFSF15* only with the whole blood data from the Genotype-Tissue Expression (GTEx) project, highlighting the utility of building a population-specific data set, even of modest size. The risk alleles of these genes were found to be associated with lower expression levels of *TNFSF15* and *GPR35*, respectively. Our eQTL browser can be accessed at “http://asan.crohneqtl.com/”.

**Conclusion:**

This resource would be useful for studies that need to employ genome-wide association analyses involving Asian populations.

## Introduction

Genome-wide association studies (GWAS) have identified >200 loci that are associated with inflammatory bowel diseases (IBD) (Liu et al., 2015; de Lange et al., 2017). As GWAS variants do not necessarily identify causal genes or reveal how the variants influence traits, the effects of GWAS variants on diseases remain largely unknown. Most IBD associated variants are in the non-coding regions of the genome, suggesting that the variants may affect the trait through regulation of gene expression. Expression quantitative trait locus (eQTL) studies associate genomic and transcriptomic data sets from the same individuals to identify loci that affect mRNA expression. By linking SNPs to changes in gene expression, eQTL can be useful for annotating GWAS variants. Recently, several methods have been proposed to connect eQTL data to GWAS associations in an integrative framework often referred to as transcriptome-wide association studies (TWAS) (Gusev et al., 2016; Zhu et al., 2016). The statistical framework “eQTL and GWAS CAusal Variants Identification in Associated Regions” (eCAVIAR) estimates the probability that the same variant can be causal for both expression changes and disease status (Hormozdiari et al., 2016). Summary-data-based Mendelian Randomization (SMR) integrates GWAS with eQTL data by testing if the eQTL occurs to be associated with the disease (Zhu et al., 2016). These methods allow determination of the most functionally relevant genes at the loci and identification of novel trait-associated loci via imputation of genotype correlated gene expression levels into GWAS datasets (Gusev et al., 2016; Zhu et al., 2016). Using such methods, recent studies have suggested that a large number of candidate common trait susceptibility genes identified as eQTL are not the nearest genes to the GWAS lead SNPs, suggesting that causal genes might be distinct from the nearest genes (Hormozdiari et al., 2016). Regulatory elements can act over a long distance and in a cell-type specific manner, making the identification of the causal genes for a given pathologic condition and their roles extremely difficult.

Recently, eQTL have been identified using a broad collection of normal human tissues from diverse populations, increasing our understanding of regulation of gene expression. However, non-European ancestries are under-represented in the current eQTL databases. Comparisons of patterns of gene expression between European and African populations have revealed that more distantly related populations tend to have a greater number of differentially expressed genes, which often result from expression of different genes rather than differences in the expression levels of the same genes (Lappalainen et al., 2013). Studies on the genetic architecture of gene expression traits across three different populations found that genetic correlation of gene expression depends on shared ancestry proportions, emphasizing that a training set with ancestry similar to the test set is better at predicting gene expression in the test populations (Mogil et al., 2018).

The most recent GWAS on Asian patients with Crohn’s disease (CD) has reported 29 susceptibility loci (Yang et al., 2016); however, integration of disease association and eQTL analyses for Asian CD has never been conducted before. To determine the most functionally relevant genes at the CD loci identified in Asian GWAS and to find novel CD-associated loci, we built a Korean-CD-specific eQTL data set from whole blood of CD patients. We performed eQTL analysis using RNA-seq of whole blood in a cohort of 101 Korean CD patients and tried to integrate with the Korean CD GWAS to identify candidate causal variants at two known loci. We found not only substantial overlap between our eQTL and the blood eQTL database on a very large cohort but also additional eQTL unique to our study population. As the eQTL database was constructed using CD patients and not healthy individuals, Korean CD eQTL datasets might provide a valuable resource for link between genetic variation and gene expression and regulation not only in Asians, but also in Caucasians if new eQTL in patients become only evident when the gene is overexpressed as a result of modified inflammatory status.

## Materials and Methods

### Study Subjects

A total of 101 CD patients were recruited from the IBD Clinic of Asan Medical Center. All these study subjects provided written informed consent. The clinical characteristics of the 101 CD patients are shown in Supplementary Table S1. These patients had previously been genotyped as part of a previous GWAS (Yang et al., 2016), and their genotype data were included in the eQTL analysis of the study presented here.

### Genotype Quality Control and Imputation

Quality control (QC) was performed as previously described GWAS (Yang et al., 2016). Briefly, individuals with a call rate ≤96% were removed. We excluded all the single nucleotide polymorphisms (SNPs) with missing rates >0.02, minor allele frequency <0.01, and Hardy-Weinberg equilibrium (HWE, *P* < 1.0 × 10^–5^). After the QC, we had 101 individual samples and 524,635 genotyped SNPs available for the downstream eQTL analyses.

To increase the power for eQTL discovery, the SNPs that passed QC were further subjected to haplotype phasing and genotype imputation using SHAPEIT (Delaneau et al., 2011) and IMPUTE2 (Howie et al., 2009), respectively, based on the 1000 Genome multi-ethnic reference panel (Feb 2012, IMPUTE v2). The imputed genotypes were subjected to a second round quality control with the imputed variants (info >0.8), missing rates <0.02, MAF > 0.01, and HWE *P* > 1.0 × 10^–5^ using SNPTEST (Marchini et al., 2007). In total, 6,451,113 SNPs including 524,635 genotyped and 5,926,478 imputed SNPs were retained.

### Total RNA Extraction From the Peripheral Blood, and Library Preparation and Sequencing

Total RNA was isolated from the peripheral blood using PAXgene Blood RNA system (PreAnalytiX, QIAGEN, Germany). Whole blood was taken and immediately store in a PAXgene Blood RNA tube at room temperature for >4 h. The total RNA was extracted using the PAXgene Blood RNA kit, following the manufacturer’s instructions. RNA quality and quantity were checked using a 2100 Bioanalyzer (Agilent Technologies, CA, United States) and the samples with an RNA integrity number ≥7 were deep-sequenced. Sequencing libraries were prepared with the Illumina TruSeq Stranded Total RNA Library Prep Kit with Ribo-Zero Globin (Illumina, CA, United States) and paired-end RNA sequencing of 101 bp reads was performed using Illumina HiSeq 2500 platform.

### Quality Validation and Alignment

Paired-end RNA sequencing of 2 × 101 bp reads was performed using Illumina HiSeq 2500 platform. We evaluated the number and quality of the total reads, GC percent and adapters in the raw fastq files using FastQC v0.11.7^1^. Every one of the 101 samples had >93 million raw reads (117 million reads on average) and passed the read quality check (average Phred quality score >36), and GC percent check (46-56%). The adapter sequences and reads with low quality were excluded using cutadapt (Martin, 2011) applying the quality Phred score cut-off <33 and read length cut-off <20 bp. We performed alignment for the trimmed reads using STAR (default settings) (Dobin et al., 2013) and GRCh37 reference genome in GENCODE release 19^2^ (Frankish et al., 2019). For confirmation of unique mapping rate and ribosomal RNA rate, we used RNA-SeQC (DeLuca et al., 2012). As 15 CD patients showed high ribosomal RNA ratio (>40%) in sample QC, the RNA sequencing of these 15 samples were repeated and aligned to the reference genome. We confirmed high unique mapping rate (94-99%) in all the 101 samples. After alignment, we used RNA-SeQC to estimate the transcript abundance, expected read counts, and transcripts per million reads (TPM) for each gene by selecting the uniquely mapped reads with a mapping quality >255, and ≤6 mismatched bases to the reference genome.

### eQTL Analysis

After removing low-expressed genes from the mRNA expression data estimated by RNA-SeQC, 21,718 genes with TPM > 0.1 and the number of reads >6 in ≥20% of the 101 CD samples were included. With the read count data of the 21,718 genes from the 101 samples, we generated a multi-dimensional scaling (MDS) plot using an R package: edgeR^3^ (Robinson et al., 2010) to confirm absence of batch effect in the RNA sequencing data from 15 samples that had been re-sequenced due to high rRNA contamination (Supplementary Figure S1). We used a trimmed mean of *M*-values (TMM) for normalization of gene expression values considering total mRNA read counts of each sample using edgeR (Robinson et al., 2010). The genomic input data of the 101 CD patients included a total of 6,451,113 SNPs from GWAS. The *cis* window was defined as the 1-Mb region up- and downstream of the transcription start site (TSS) (±1 Mb). Dosage was used for the association analysis for imputed SNPs. Nominal *P* values were calculated for each SNP-gene pair with FastQTL (Ongen et al., 2016) using the linear regression model with 27 covariates including 15 PEER (Stegle et al., 2012) factors, 3 PCs calculated using GWAS dataset of 101 CD samples, repeat or not, gender, age, age of diagnosis, follow-up year, family history, smoking or not, Montreal classification, and disease behaviors. Significance of the top associated variant per gene was estimated by adaptive permutation with the setting “–permute 1000 10000” in FastQTL (Ongen et al., 2016). The beta distribution-adjusted empirical *P* values were used to calculate the *q*-values and FDR thresholds of each gene using R package: *q* value^4^. The FDR threshold of <0.05 was applied to identify all the significant *cis-*eQTL.

### Enrichment Analysis on eGene Set

To annotate the biological mechanisms related to the eGenes in the eQTL analysis of the 101 CD patients, we performed the Gene Ontology (GO) (Ashburner et al., 2000; The Gene Ontology Consortium, 2017) enrichment analysis in the web application, AmiGO2^5^ (Carbon et al., 2009) using 3,816 eGenes with FDR < 0.05 in the *cis-*eQTL analysis. By the default setting (GO aspect: biological process, Species: Homo sapiens), the result page showed the over- or underrepresented GO terms with significant *P* values.

### Comparison of the Direction of Allelic Effects Across the Existing *Cis-*eQTL Databases

We compared the allelic directions of SNP-gene associations shared among the Korean CD *cis-*eQTL, the existing whole blood *cis-*eQTL databases of Japanese (105 healthy individuals)^6^ (Ishigaki et al., 2017), and GTEx V7 (369 individuals)^7^ (Aguet et al., 2017). The number of *cis-*eQTL in the Korean CD, Japanese, and GTEx datasets was 135,164, 335,813 and 1,052,542, respectively. Using only significant *cis-*eQTLs with *q* value ≤ 0.05 in each dataset, we compared the slope of the overlapping SNP-gene associations between three pairs of Korean CD-Japanese, Korean CD-GTEx, and GTEx-Japanese datasets.

### Colocalization Analysis Between CD GWAS and Whole Blood eQTL

For colocalization analysis between CD GWAS of 899 cases and 3,805 controls (Yang et al., 2016) and whole blood eQTL data of 101 CD patients, we used eCAVIAR (Hormozdiari et al., 2016) to estimate the probability of eQTL and GWAS sharing the same causal variants. The association test of CD GWAS was performed by frequentist association test of SNPTEST (Marchini et al., 2007) using the additive model. The eCAVIAR calculated co-localization posterior probability (CLPP) score, indicating the level of colocalization, using each Z score of eQTL and GWAS data, as well as linkage disequilibrium (LD) information. We used the LD reference of East Asians (JPT + CHB) in the 1000 genomes^8^ (May 2015 release). We also tried Japanese eQTL and GTEx eQTL datasets for colocalization analysis with CD GWAS. For GTEx, LD reference of Caucasian (CEU) in the 1000 genomes was used since eCAVIAR allows different LD structures for eQTL and GWAS datasets. We selected 100 SNPs upstream and downstream of the reported lead SNPs in the 29 established loci (excluding the major histocompatibility complex (MHC) region, 25 ∼ 34 Mb) in Koreans (Yang et al., 2016; Lee et al., 2018) to calculate the CLPP score. We used the default of two causal variants for locus and eCAVIAR method’s recommended significant cut-off, CLPP > 0.01, and 0.95 for total credible set posterior probability.

### SMR and HEIDI Test

We performed a transcriptome-wide association study (TWAS) integrating CD GWAS of 4,704 individuals with the expression data from the whole blood tissue of the 101 CD patients using SMR software^9^ (Zhu et al., 2016). We used associations from SNPTEST (Marchini et al., 2007) result of the previously reported GWAS including 899 CD cases and 3,805 controls, and the whole blood *cis-*eQTL of the 101 CD patients as the input data of SMR and heterogeneity in dependent instruments (HEIDI) test. LD was estimated using the individual genotype data of East Asians (JPT + CHB) in the 1000 genomes (see text “footnote 8”, May 2015 release) after excluding the MHC region (25 ∼ 34 Mb) due to LD complexity. Excluding all the genes with *P*_*HEIDI*_ < 0.05 in the HEIDI test for detection of heterogeneity, we selected the target genes with a shared causal variant between the GWAS and *cis-*eQTL data applying a threshold of *P*_*SMR*_ < 2.3 × 10^–6^ (0.05/21,583 genes). We also performed a TWAS integrating the CD GWAS of 4,704 individuals with Japanese eQTL using SMR.

## Results

### *Cis-*eQTL

To identify the *cis-*eQTL variants within 1 Mb on either side of the TSS of each gene, *cis-*eQTL analysis was performed using the GWAS and RNA sequencing data from the peripheral blood of 101 Korean CD patients. Applying the threshold of FDR < 0.05, we found 135,164 *cis-*eQTL, 104,900 eSNPs, and 3,816 eGenes which had at least one *cis-*eQTL. The distance from an eSNP to the TSS of the target gene was ≤500 kb in 95.7% (129,333 *cis-*eQTL) and ≤250 kb in 86.9% (117,433 *cis-*eQTL) of the total 135,164 *cis-*eQTL, and locations of eSNPs were more likely to be near the TSS of their target genes (Supplementary Figure S2). Of the total 104,900 eSNPs, the number of the target genes was one for 83,848 eSNPs (79.9%), two for 15,508 eSNPs (14.8%), and over three for 5,544 eSNPs (5.3%) (Table 1). The proportion of the eSNPs to the total SNPs in each chromosome was 0.6-3.5% (total = 1.6%), and the ratio of the eGenes to the total genes ranged from 14.4 to 23.0% (total = 17.6%). The gene biotypes of 3,816 eGenes were composed of 2,700 protein coding genes (70.8%), 418 pseudogenes (11.0%), 272 antisense RNAs (7.1%), 270 long intergenic non-coding RNAs (7.1%), 44 sense intronic non-coding RNAs (1.2%), and 43 processed transcripts (1.1%), and 69 other gene biotypes (1.8%) (Supplementary Figure S3).

**TABLE 1:** The number of *cis-*eQTLs, eSNPs and eGenes in each chromosome.

CHR	*Cis-*eQTLs	GWAS SNPs			eSNPs					eGenes		
		Genotyped Genotyped	Imputed	Total	l eQTL	2 eQTLs	>3 eQTLs	Total	Proportion (%)^a^	Genes	eGenes	Proportion (%)^b^
1	11,420	43,125	460,009	503,134	8,178	926	378	9,482	1.9	2,280	360	15.8
2	12,314	42,445	492,868	535,313	7,395	947	1,006	9,348	1.7	1,580	287	18.2
3	7,487	35,633	434,092	469,725	4,931	1,161	78	6,170	1.3	1,296	210	16.2
4	6,768	30,082	448,317	478,399	4,218	341	456	5,015	1.0	854	174	20.4
5	6,341	31,540	383,596	415,136	3,777	811	310	4,898	1.2	1,043	192	18.4
6	15,859	35,863	427,405	463,268	9,345	2,644	403	12,392	2.7	1,066	224	21.0
7	7,180	28,274	355,223	383,497	4,331	1,145	156	5,632	1.5	1,083	216	19.9
8	3,781	27,460	327,106	354,566	2,968	345	40	3,353	0.9	806	158	19.6
9	3,376	24,614	259,305	283,919	2,290	366	98	2,754	1.0	916	141	15.4
10	8,172	28,916	308,710	337,626	5,568	1,126	86	6,780	2.0	842	157	18.6
11	6,204	27,091	294,143	321,234	4,382	803	72	5,257	1.6	1,220	196	16.1
12	11,754	26,471	287,778	314,249	4,257	977	1,184	6,418	2.0	1,194	255	21.4
13	1,633	20,487	217,333	237,820	1,182	218	5	1,405	0.6	392	66	16.8
14	3,997	17,482	194,929	212,411	2,756	481	93	3,330	1.6	817	133	16.3
15	4,507	16,715	165,539	182,254	2,557	641	222	3,420	1.9	769	136	17.7
16	4,254	16,922	171,964	188,886	2,262	578	184	3,024	1.6	1,054	168	15.9
17	6,265	14,941	143,621	158,562	3,929	724	245	4,898	3.1	1,329	211	15.9
18	1,846	16,131	164,466	180,597	1,274	112	116	1,502	0.8	350	68	19.4
19	5,366	10,993	119,710	130,703	4,050	266	219	4,535	3.5	1,438	222	15.4
20	2,183	13,804	122,336	136,140	1,881	151	0	2,032	1.5	556	80	14.4
21	1,381	7,919	78,373	86,292	754	180	81	1,015	1.2	256	59	23.0
22	3,076	7,727	69,655	77,382	1,563	565	112	2,240	2.9	577	103	17.9
Total	135,164	524,635	5,926,478	6,451,113	83,848	15,508	5,544	104,900	1.6	21,718	3,816	17.6

*CHR, chromosome; eQTL, expression quantitative trait loci; GWAS, genome-wide association study; SNP, single nucleotide polymorphism. ^*a*^ Proportion of the eSNPs per chromosome GWA-SNPs. ^*b*^ Proportion of eGenes in total number of genes used for eQTL analysis per chromosome.*

To annotate the biological processes significantly related to the 3,816 eGenes, we performed GO enrichment analysis using web-based AmiGO2 (see text “footnote 5”) (Carbon et al., 2009). Of those, 1,051 eGenes were excluded from the analysis mainly due to being non-coding genes or being absent in the reference genes of the GO dataset. A total of 2,765 eGenes were used as the input gene list. Of the significantly shared GO terms with the Bonferroni-corrected *P* value < 0.05, granulocyte activation (GO:0036230) and neutrophil activation (GO:0042119) showed the top two highest fold enrichment values, respectively (Supplementary Table S2).

### Comparison of Direction of Allelic Effects Across Existing *Cis-*eQTL Databases

**FIGURE 1:**
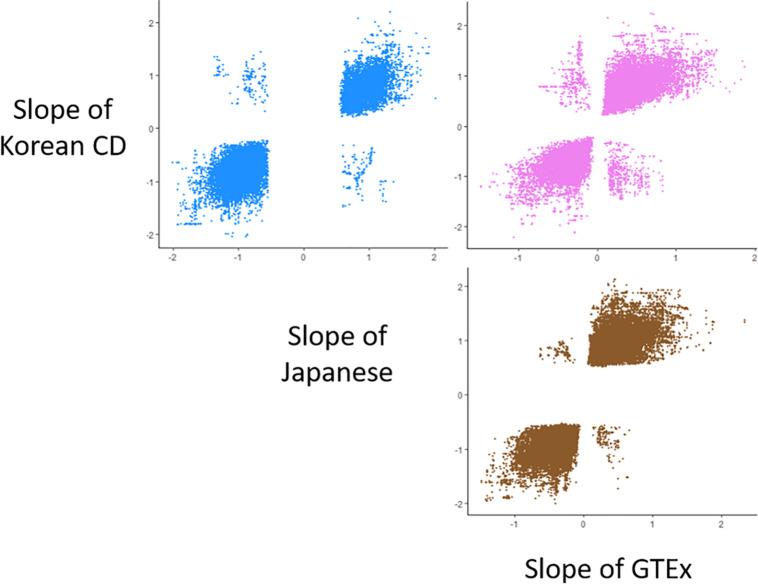
Scatter plots for comparison of the direction of allelic effects among the whole blood *cis-*eQTL data from the Korean CD, Japanese samples, and GTEx project. Each point on the scatter plots represents the allelic effect of a SNP to a gene expression. The scatter plots included 50,848 *cis-*eQTLs of 1,201 eGenes between the Korean CD and Japanese samples, 58,197 *cis-*eQTLs of 1,581 eGenes between the Korean CD and GTEx, and 120,158 *cis-*eQTLs of 1,873 eGenes between the GTEx and Japanese samples.

Using the Korean CD eQTL data and two existing *cis-*eQTL datasets derived from whole blood samples, including the Japanese eQTL (Ishigaki et al., 2017) and GTEx (Aguet et al., 2017), we compared the direction of allelic effects of all the common SNP-gene pairs. Of the 135,164 eQTL in the Korean CD, 335,813 in the Japanese, and 1,052,542 in the GTEx datasets, the number of shared significant *cis-*eQTL (a threshold of *q* value ≤ 0.05) in each pair was 50,848 between the Korean CD and Japanese eQTL, 58,197 between the Korean CD and GTEx, and 120,158 between the GTEx and Japanese eQTL datasets (Figure 1). In total, 96.5–98.7% of shared eGenes in each pair of the three *cis-*eQTL datasets showed the same direction of allelic effects. The proportion of shared eGenes with the opposite direction of allelic effects was 16 of 1,201 (1.3%) shared eGenes in the pair of Korean CD-Japanese, 44 of 1,873 (2.3%) shared eGenes in the pair of GTEx-Japanese *cis-*eQTL datasets and 56 of 1,581 (3.5%) shared eGenes in the pair of Korean CD-GTEx (Supplementary Table S3). Between the 16 and 56 eGenes with the opposite direction of allelic effects in the pair of Korean CD-Japanese or -GTEx, 9 eGenes overlapped. Of the SNPs with top eQTL *P* value of the 9 eGenes, only rs2859102 in *TAP2* showed significant association with CD in Koreans (*P* = 8.81 × 10^–11^).

### Identification of CD Susceptibility Genes From GWAS Loci Using Whole Blood eQTL

**FIGURE 2:**
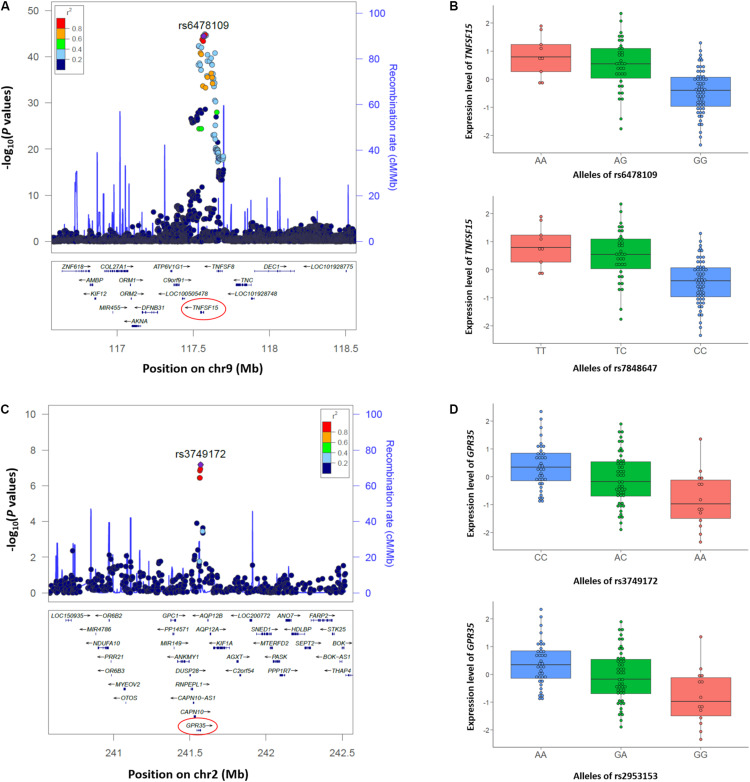
Two loci including *TNFSF15* and *GPR35* identified with colocalization analysis between CD GWAS and *cis-*eQTL data from the Korean CD samples. **(A,C)** Regional association plots of the **(A)** *TNFSF15* locus at 9q32 using 5,956 SNPs, and **(C)** *GPR35* locus at 2q37 using 497 SNPs in ±1 Mb from rs6478109 and rs3749172 are plotted according to their chromosomal positions (hg19) with –log_10_ *P* values from the Korean CD GWAS. Both rs6478109 and rs3749172 are shown as purple circles in each plot. LD (r2) indicated with colors was calculated using East Asian population data (JPT + CHB) for the 1000 Genomes (http://www.internationalgenome.org). Regional association plots were generated using a web browser, LocusZoom (http://locuszoom.org/genform.php?type=yourdata). **(B,D)** Box plots of the **(B)** *TNFSF15* expression level according to alleles of rs6478109 and rs7848647, and **(D)** *GRP35* expression level according to alleles of rs3749172 and rs2953153. Small circles in the box plot indicate the normalized expression level using trimmed mean of *M*-values (TMM).

We also performed colocalization analyses of the CD GWAS and whole blood eQTL data of the Korean CD, Japanese, and GTEx datasets using eCAVIAR (Hormozdiari et al., 2016). eCAVIAR computes the CLPP score using Z statistics of GWAS and *cis-*eQTL data of 100 SNPs upstream and downstream of the reported lead SNP in the 29 established loci in Koreans (Yang et al., 2016; Lee et al., 2018). We identified 122 eGenes within 1 Mb window from the lead SNP in the 29 established loci as target genes for *cis-*eQTL data of the Korean CD dataset. Applying thresholds of CLPP > 0.01 and total credible set posterior probability >0.95, two loci including *TNFSF15* (TNF Superfamily Member 15) at 9q32 and *GPR35* (G-protein coupled receptor 35) at 2q37 were identified in colocalization analysis between the GWAS and *cis-*eQTL data of the Korean CD dataset (Table 2 and Supplementary Table S4). In the *TNFSF15* locus, a previously reported SNP (rs6478109) (Yang et al., 2016) at 360 bp upstream of *TNFSF15* (Figure 2A) showed a significant *P* value in both GWAS (3.2 × 10^–45^) and *cis-*eQTL (3.6 × 10^–10^) with a CLPP score of 0.23. The other causal SNP, rs7848647 at 640 bp upstream of *TNFSF15* is in complete LD (*r*^2^ = 1) with rs6478109 in East Asians. Both risk allele G of rs6478109 and risk allele C of rs7848647 in CD GWAS were related to lower expression of *TNFSF15* than each protective allele A or T in the whole blood tissue (Figure 2B). A reported SNP in the *GPR35* locus, rs3749172 (p.Ser294Arg) (Liu et al., 2015; Yang et al., 2016) is located in exon 6 and 25,401 bp downstream of the TSS of *GPR35* (Figure 2C). The CLPP score (0.17) of rs3749172 showed that rs3749172 was a shared causal variant in CD GWAS (*P* value = 6.7 × 10^–8^) and whole blood *cis-*eQTL (*P* value = 4.4 × 10^–6^) (Table 2). The other causal SNP, rs2953153 (CLPP = 0.20) in intron 5 and 21,164 bp downstream of the TSS of *GPR35*, is in high LD (*r*^2^ = 0.98) with rs3749172. Expression of *GPR35* in whole blood was down-regulated at risk allele A of rs3749172 and risk allele G of rs2953153, but up-regulated at protective allele C and A, respectively (Figure 2D).

**TABLE 2:** Colocalization analysis between CD GWAS and whole blood eQTL using eCAVIAR in *TNFSF15* and *GPR35* locus.

Locus	Gene	SNP	Position (hg19)	Allele		Credible set posterior probability^*a*^	CLPP^*b*^	CD GWAS^*c*^				Whole blood -*cis* eQTL		
				Risk	Non-risk			RAF Cases	RAF Controls	*P* value	OR	*P* value^*d*^	Slope^*e*^	Distance (BP)^*f*^
9q32	*TNFSF15*	rs6478109	117,568,766	G	A	0.50	0.23	0.706	0.521	3.19 × 10^–45^	2.10	3.59 × 10^–10^	−0.75	360
		rs7848647	117,569,046	C	T	0.50	0.23	0.644	0.513	3.29 × 10^–^*^45^*	2.10	3.59 × 10^–10^	−0.75	640
2q37	*GPR35*	rs2953153	241,566,012	G	A	0.51	0.20	0.362	0.297	1.38 × 10^–7^	1.34	4.40 × 10^–6^	−0.61	21,164
		rs3749172	241,570,249	A	C	0.45	0.17	0.361	0.295	6.65 × 10^–8^	1.34	4.40 × 10^–6^	−0.61	25,401

*BP, base pair; CD; Crohn’s disease; CLPP, colocalization posterior probability; eQTL, expression quantitative trait loci; GWAS, genome-wide association study; OR, odds ratio; RAF, risk allele frequency; SNP, single nucleotide polymorphism. ^*a*^Posterior probability of each causal variant within the credible set (threshold >0.95). ^*b*^Colocalization posterior probability indicates the level of colocalization (threshold >0.01). ^*c*^Frequentist association test of 899 CD and 3,805 controls using SNPTEST. ^*d*^eQTL *P* value calculated using FastQTL. ^*e*^Effect size of gene expression levels of risk versus non-risk alleles. ^*f*^Distance from the SNP position to the transcription start site of the gene.*

In the colocalization analysis of the 118 target genes between the CD GWAS and Japanese *cis-*eQTL data, we only identified a *TNFSF15* locus with two causal SNPs, rs6478109 and rs7848647 (CLPP score = 0.13, total credible set posterior probability = 1), above the threshold (Supplementary Table S5). In the *GPR35* locus, rs2953153 and rs3749172 had significant CLPP scores of 0.10 and 0.39, respectively; however, a total of credible set posterior probabilities of rs2953153 and rs3749172 were below the threshold of 0.89. The colocalization analysis of the 348 target genes between the CD GWAS and GTEx identified rs6478109 and rs7848647 at the *TNFSF15* locus as the most likely causal SNP, distinct from the European lead SNP rs10114470 (*r*^2^ = 0.77 with rs6478109) in the largest CD GWAS (de Lange et al., 2017; Supplementary Table S6). These two causal SNPs showed significant CLPP scores (rs6478109 = 0.06 and rs7848647 = 0.09) and a 100% credible set posterior probability. Both rs2953153 and rs3749172 in the *GPR35* locus had non-significant CLPP scores < 0.01 and *cis-*eQTL *P* values with FDR > 0.05 for *GPR35* expression. European lead SNP in the largest CD GWAS was rs34236350 (de Lange et al., 2017), in moderate LD with the Asian lead SNP rs3749172 (*r*^2^ = 0.34) in Europeans. The colocalization analysis of the *GPR35* locus between the CD GWAS of European ancestry (de Lange et al., 2017) and GTEx failed to identify shared causal SNPs.

### Transcriptome-Wide Association Study (TWAS) Using Whole Blood eQTL Data

Using summary statistics of the CD GWAS of 4,704 individuals and whole blood *cis-*eQTL data of the 101 CD patients, TWAS was performed using SMR (Zhu et al., 2016) to discover genes whose expression levels were related to the causal variants of the CD GWAS. Of the top 21 CD TWAS genes at 18 loci (*P*_*SMR*_ < 0.01 and *P*_*HEIDI*_ > 0.05), 5 TWAS genes were at four previously known CD GWAS loci (Supplementary Table S7). Applying the threshold of *P*_*HEIDI*_ > 0.05 and *P*_*SMR*_ < 2.31 × 10^–6^ (0.05/21,583 genes), SMR test identified only *TNFSF15* at 9q32 with *P*_*SMR*_ = 8.07 × 10^–9^ and *P*_*HEIDI*_ = 0.13 (Supplementary Figure S4). A lead SNP of TWAS in the *TNFSF15* locus was rs6478108 in intron 1 of *TNFSF15* with *P* value = 1.6 × 10^–44^ in the CD GWAS, and the most significant *P* value in *cis-*eQTL (2.47 × 10^–10^) for *TNFSF15* expression (Supplementary Table S7). rs6478108 is in high LD (*r*^2^ = 0.98) with rs6478109 discovered in the colocalization analysis, and risk allele T of rs6478108 in the CD GWAS was associated with lower expression of *TNFSF15* than allele C in the whole blood of the CD patients. When we performed TWAS using Japanese eQTL, SMR test identified only *TNFSF15* at 9q32 with *P*_*SMR*_ = 5.95 × 10^–10^ and *P*_*HEIDI*_ = 0.2 (Supplementary Table S8). Between the top 27 genes at 22 loci and 21 genes at 18 loci (*P*_*SMR*_ < 0.01 and *P*_*HEIDI*_ > 0.05) identified using the Japanese eQTL and Korean CD eQTL, respectively, 10 loci (11 genes) overlapped (Supplementary Tables 7, 8).

## Discussion

In this study, we established a whole blood eQTL dataset from Korean patients with CD. Our dataset identified eQTL unique to our study population and also confirmed the eQTL signals previously identified in other populations. Using this dataset, we further identified putative target genes at two previously reported loci. We also tried to identify CD-associated genes by TWAS.

Although the eQTL signals identified in other populations were consistent with that identified in our samples, and 96.5-98.7% of the shared eGenes in each pair of three *cis-*eQTL datasets showed the same direction of allelic effects, we were able to colocalize the *GPR35* locus using only the Korean CD or Japanese eQTL, but not GTEx whole blood. Colocalization analysis with the Korean CD or Japanese eQTL identified the same SNPs in the *TNFSF15* and *GPR35* loci, although the *GPR35* locus was less significant in the Japanese eQTL, whereas colocalization analysis of the Korean CD GWAS and GTEx whole blood identified *TNFSF15* only. Of note, *TNFSF15* risk allele was found to be associated with decreased expression in the whole blood of Koreans, consistent with a recent finding involving a European population (Richard et al., 2018). The top signals of Korean and European CD GWAS at the *GPR35* locus were rs3749172 and rs34236350, respectively, in moderate LD (*r*^2^ = 0.34) in Caucasians while in high LD in Asians. Rs34236350 of European CD GWAS signal was eQTL for *GPR35* in GTEx sigmoid colon tissue only with up-regulation at risk allele, which is in the opposite direction relative to the whole blood eQTL of Korean and Japanese populations. Due to non-significant expression of *GPR35* in GTEx whole blood (FDR > 0.05), colocalization using GTEx did not identify *GPR35*, indicating that population-specific eQTL effects exist. Therefore, our data highlight the utility of building a population-specific data set, even of modest size.

We found that the most significantly enriched GO terms of the 3,816 eGenes was granulocyte activation, especially neutrophil degranulation. The role of neutrophils in the pathogenesis of CD has been much better described in a theory that the common predisposition to CD is a failure of the inflammatory response to tissue damage and innate immunity (Segal, 2019). Failure of neutrophil migration to the inflammatory site is one of the mechanisms involved in granulomatous inflammation, characteristic of CD, which leads to an intense adaptive immune response and the tissues become infiltrated with large number of T cells. These cells as well as macrophages will react by producing cytokines that cause local inflammation and systemic symptoms. The GO term of the two genes identified through colocalization analysis, *TNFSF15* and *GPR35*, was cellular process (GO:0009987) (Supplementary Table S2). GPR35, the receptor of the mucosal chemokine CXCL17 (Maravillas-Montero et al., 2015), is expressed in a subset of macrophages that are recruited to mucosal tissues by CXCL17 (Hernández-Ruiz and Zlotnik, 2017). Given the importance of macrophages in inflammation and the strong expression of both CXCL17 and GPR35 in mucosal tissues, down-regulated at CD risk allele of GPR35 can cause the failure of recruitment of myeloid cells to the site of inflammation. TNFSF15, a proinflammatory cytokine (Jin et al., 2013), can stimulate CSF2 (Colony Stimulating Factor 2) expression which can promote proliferation and maturation of granulocytes (Bhattacharya et al., 2015). Given *TNFSF15* risk allele was associated with decreased expression, which can result in decrease in neutrophil activation level.

We recognize several limitations of our study. First, because of our small sample size, we had limited statistical power to detect rare or low impact eQTL, and thus we may have underestimated the number of genes overlapping with those reported in other studies. Additionally, the rather limited GWAS sample size did not allow our TWAS to identify novel candidate genes above the threshold. Second, we assessed gene expression in the whole blood of CD patient, whereas Japanese and GTEx whole blood eQTL were from healthy individuals. The Korean CD eQTL colocalized with the CD GWAS variants at two known loci, but at only one known locus with the Japanese eQTL. TWAS using the Korean CD and Japanese eQTL identified 21 and 27 genes at 18 and 22 loci (*P*_*SMR*_ < 0.01 and *P*_*HEIDI*_ > 0.05), respectively. TWAS using both eQTLs identified *TNFSF15* only above the threshold; however, there were differences in the additional genes identified: TWAS using the Korean CD eQTL identified *GPR35*, whereas TWAS using the Japanese eQTL identified *RNASET2*. These differences might be due the differences in gene expression between the CD patient and control blood samples and/or population differences between Korean and Japanese. Although whole blood gene expression of the CD patients and healthy individuals show a high degree of similarity, we cannot exclude the possibility that new eQTL in patients become only evident when the gene is over-expressed as a result of modified inflammatory status. Third, we assessed gene expression in whole blood, which contains a heterogeneous population of cells and cannot be considered to be disease-relevant context, contributing to the low yield of causal genes identified for CD using the GWAS-eQTL integration approach. Although whole blood eQTL have been reported to be enriched with autoimmune disorder – associated SNPs (GTEx Consortium, 2015), whole blood consists of multiple distinct cell types with specific gene regulatory profiles as shown by eQTL of isolated different blood cell types (Fairfax et al., 2012). Previous GWAS have highlighted the importance of T cells in CD pathology. A recent study of single-cell RNA sequencing of blood and ileal T cells from patients with CD has shown that quiescent T cells dominate the peripheral blood (PBL) whereas cytotoxic T lymphocytes and T-helper 17 (Th 17) cells dominate intraepithelial T lymphocytes (IELs) and lamina propria T lymphocytes (LPLs), respectively (Uniken Venema et al., 2019). The authors have also noted that IELs and LPLs express significantly more CD-risk genes, whereas PBLs are not enriched for CD-risk gene expression, emphasizing importance of eQTL of disease-relevant cells.

## Conclusion

In conclusion, we made the results of our eQTL study involving Korean CD patients available. Our study confirmed the eQTL signals previously identified in other populations, while also detecting additional eQTL unique to our study population. Considering that data from non-European ancestries are limited, our dataset will be useful in furthering our understanding of pathomechanisms of CD. Our results contribute to the understanding of gene expression regulation in Koreans, and also complement existing eQTL databases in other ethnic groups. eQTL loci identified in the current study will help prioritize follow-up of findings from other GWAS of complex diseases.

## Data Availability Statement

The RNA sequencing datasets generated for this study can be found in the Gene Expression Omnibus (GEO) database GSE143507. Our eQTL browser can be accessed at “http://asan.crohneqtl.com/”.

## Ethics Statement

This study was approved by the Institutional Review Board of the Asan Medical Center. All samples were obtained with written informed consent under Institutional Review Board-approved protocols.

## Author Contributions

KS obtained financial support and conceived the study. S-KY, BY, SP, and H-SL recruited the study subjects and participated in the diagnostic evaluation. JB and JM performed the experiments. SJ and WL analyzed the data. KS, JL, and BH supervised the data analysis and interpretation. SJ and KS drafted the manuscript. WL, JL, and BH revised the manuscript.

## Conflict of Interest

BY: Grant/research support from CELLTRION, Inc.; Lecture fees from Abbvie Korea, CELLTRION, Inc., Janssen Korea, Shire Korea, Takeda Korea, and IQVIA; Consultant fees from Abbvie Korea, Ferring Korea, Janssen Korea, Kangstem Biotech, Kuhnil Pharm., Shire Korea, Takeda Korea, IQVIA, Cornerstones Health, and Robarts Clinical Trials Inc. S-KY: Grant/research support from Janssen Korea Ltd.

The remaining authors declare that the research was conducted in the absence of any commercial or financial relationships that could be construed as a potential conflict of interest.

## Footnotes

1 https://www.bioinformatics.babraham.ac.uk/projects/fastqc/

2 https://www.gencodegenes.org/human/release_19.html

3 http://bioconductor.org/packages/release/bioc/html/edgeR.html

4 https://github.com/StoreyLab/qvalue

5 http://amigo.geneontology.org/amigo

6 1https://humandbs.biosciencedbc.jp/en/hum0099-v1#hum0099.v1.eqtl.v1

7 https://gtexportal.org/home/datasets

8 http://www.1000genomes.org

9 http://cnsgenomics.com/software/smr/
