# Supplementary material for: Expression Quantitative Trait Loci (eQTL) Mapping in Korean Patients With Crohn’s Disease and Identification of Potential Causal Genes Through Integration With Disease Associations
Source: Front Genet. 2020 May 14;11:486. doi: 10.3389/fgene.2020.00486 (PMC7240107; doi:10.3389/fgene.2020.00486)
Supplement: Supplementary file 1 [file Data_Sheet_1.docx]

**SUPPLEMENTARY MATERIAL**

**Expression Quantitative Trait Loci (eQTL) mapping in Korean patients with Crohn’s disease and identification of potential causal genes through integration with disease associations.**

Seulgi Jung, Wenting Liu, Jiwon Baek, Jung Won Moon, Byong Duk Ye, Ho-Su Lee, Sang Hyoung Park, Suk-Kyun Yang, Buhm Han, Jianjun Liu, Kyuyoung Song

**Supplementary Figures 1-4 and Supplementary Tables 1-8**

Table of Contents

Supplementary Figures

Supplementary Figure 1. A multi-dimensional scaling (MDS) plot of the read count data of 21,718 high-expressed genes in 101 samples 3

Supplementary Figure 2. Location of eSNPs relative to the transcription start site of eGene 4

Supplementary Figure 3. Histogram of the gene biotypes of 3,816 eGenes 5

Supplementary Figure 4. Gene prioritization at the *TNFSF15* locus using SMR analysis 6

Supplementary Tables

Supplementary Table 1. Clinical characteristics 7

Supplementary Table 2. Gene Ontology enrichment analysis using 3,816 eGenes 8

Supplementary Table 3. Shared eGenes with the opposite direction of the allelic effect in each pair combination of the three cis-eQTL databases 9

Supplementary Table 4. Colocalization analysis between CD GWAS and whole blood cis-eQTLs of Korean CD patients using eCAVIAR (CLPP > 0.01) 11

Supplementary Table 5. Colocalization analysis between CD GWAS and whole blood cis-eQTLs of Japanese using eCAVIAR (CLPP > 0.01) 12

Supplementary Table 6. Colocalization analysis between CD GWAS and whole blood cis-eQTLs of GTEx using eCAVIAR (CLPP > 0.01) 13

Supplementary Table 7. Pairing of the eGene and lead SNPs in TWAS using CD GWAS and cis-eQTLs of Korean CD patients (*P*_SMR_ < 0.01 and *P*_HEIDI_ > 0.05) 17

Supplementary Table 8. Pairing of the eGene and lead SNPs in TWAS using CD GWAS and cis-eQTLs of Japanese (*P*_SMR_ < 0.01 and *P*_HEIDI_ > 0.05) 18


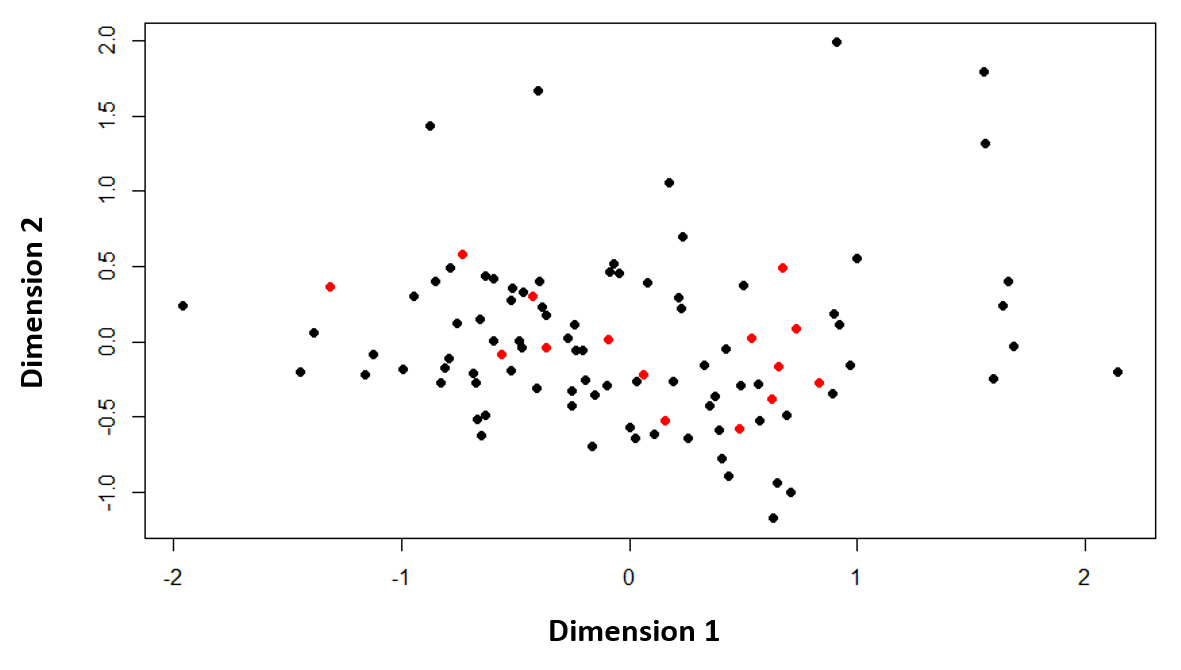


**Supplementary Figure 1. A multi-dimensional scaling (MDS) plot of the read count data of 21,718 high-expressed genes in 101 samples.** Red circles in the MDS plot represent 15 repeated samples. Distances on the plot correspond to root mean square average of the largest log_2_(fold change) between each pair of samples.


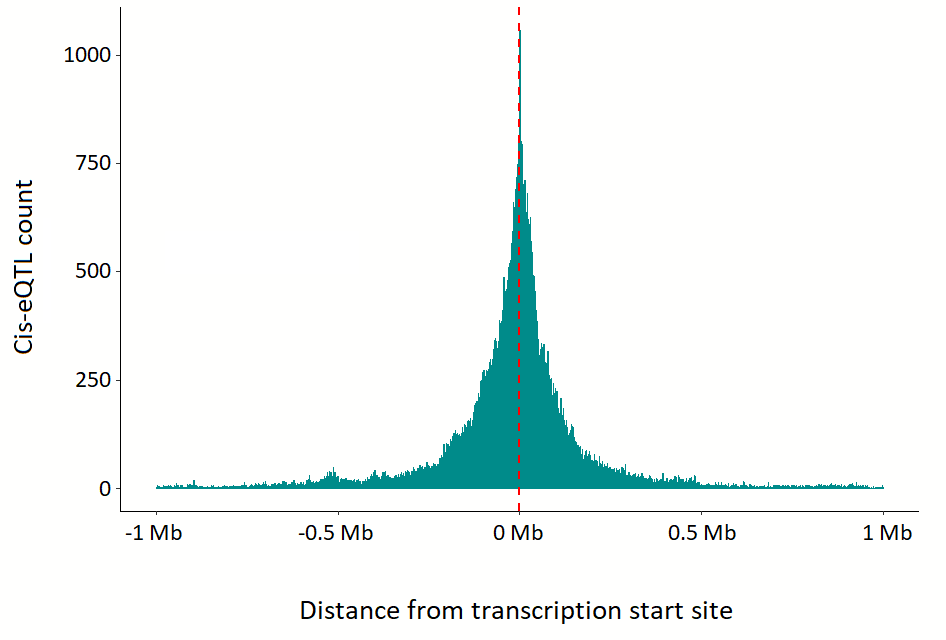


**Supplementary Figure 2. Location of eSNPs relative to the transcription start site of eGene.** The distance in histogram was determined per 1 kb bins using 104,900 eSNPs and 3,816 eGenes.


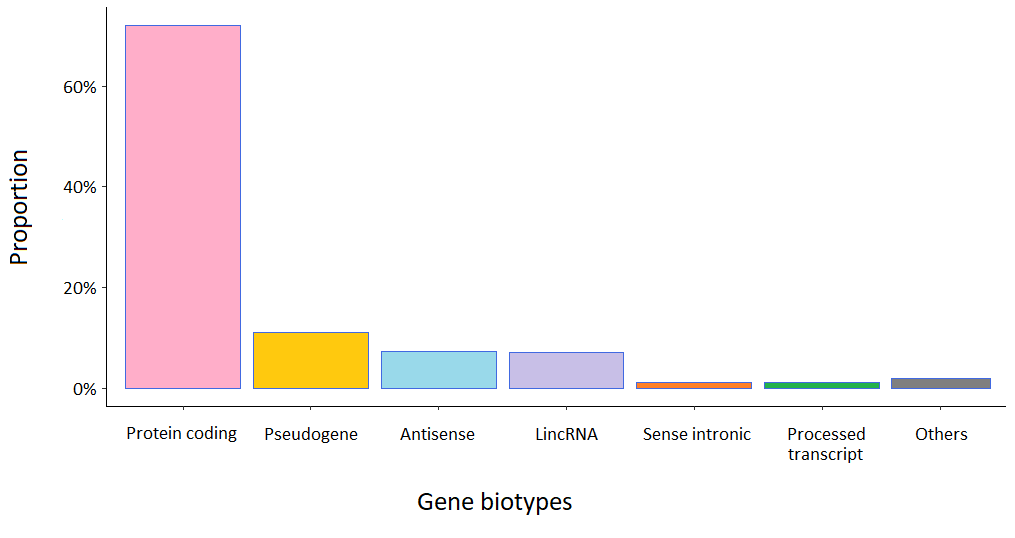


**Supplementary Figure 3. Histogram of the gene biotypes of 3,816 eGenes.** The 3,816 eGenes included 2,700 protein coding genes (70.8%), 418 pseudogenes (11.0%), 272 antisense RNA (7.1%), 270 long intergenic non-coding RNA (7.1%), 44 sense intronic non-coding RNA (1.2%), 43 processed transcript (1.1%) and 69 other gene biotypes (1.8%).


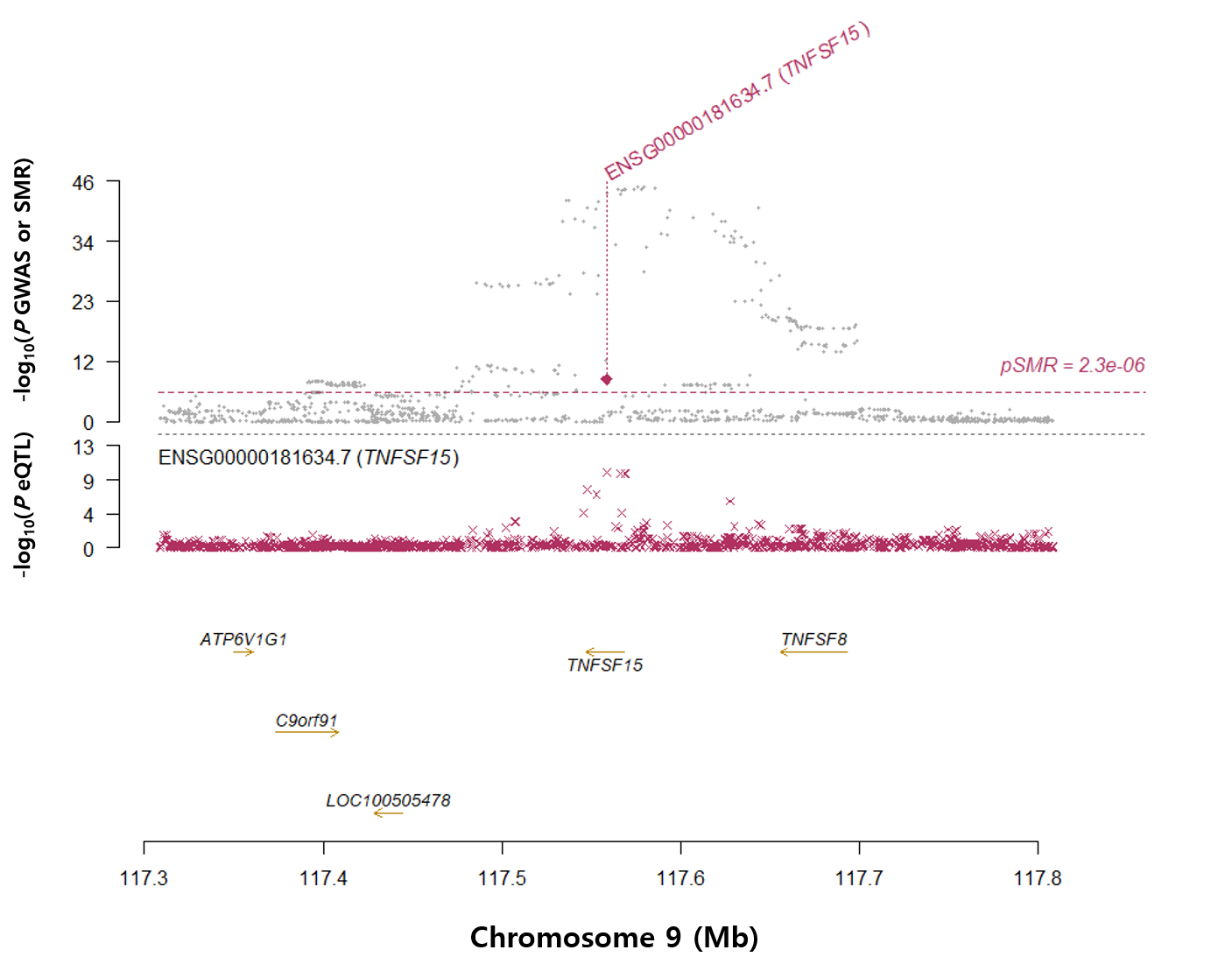


**Supplementary Figure 4. Gene prioritization at the *TNFSF15* locus using SMR analysis.** Top plot, grey dots represent association *P* values for SNPs from Korean CD GWAS and a purple diamond represents *P* value of SMR analysis between rs6478108 and *TNFSF15* expression over
